# The impact of delayed transfers of care on emergency departments: common sense arguments, evidence and confounding

**DOI:** 10.1136/emermed-2018-207917

**Published:** 2019-11-25

**Authors:** Brad Keogh, Thomas Monks

**Affiliations:** NIHR CLAHRC Wessex Data Science Hub, Faculty of Environmental and Life Sciences, University of Southampton, Southampton, UK

**Keywords:** crowding, emergency care systems, emergency departments, performance improvement, research, operational, statistics

## Abstract

**Objectives:**

There have been claims that Delayed Transfers of Care (DTOCs) of inpatients to home or a less acute setting are related to Emergency Department (ED) crowding. In particular DTOCs were associated with breaches of the UK 4-hour waiting time target in a previously published analysis. However, the analysis has major limitations by not adjusting for the longitudinal trend of the data. The aim of this work is to investigate whether the proposition that DTOCs impact the 4-hour target requires further research.

**Method:**

Estimation of an association between two or more variables that are measured over time requires specialised statistical methods. In this study, we performed two separate analyses. First, we created two sets of artificial data with no correlation. We then added an upward trend over time and again assessed for correlation. Second, we reproduced the simple linear regression of the original study using NHS England open data of English trusts between 2010 and 2016, assessing correlation of numbers of DTOCs and ED breaches of the 4-hour target. We then reanalysed the same data using standard time series methods to remove the trend before estimating an association.

**Results:**

After introducing upward trends into the uncorrelated artificial data the correlation between the two data sets increased (R^2^=0.00 to 0.51 respectively). We found strong evidence of longitudinal trends within the NHS data of ED breaches and DTOCs. After removal of the trends the R^2^ reduced from 0.50 to 0.01.

**Conclusion:**

Our reanalysis found weak correlation between numbers of DTOCs and ED 4-hour target breaches. Our study does *not* indicate that there is no relationship between 4-hour target and DTOCs, it highlights that statistically robust evidence for this relationship does not currently exist. Further work is required to understand the relationship between breaches of the 4-hour target and numbers of DTOCs.

**Key messages**

**What is already known on this subject**

- Emergency Department (ED) breaches of waiting time targets in the UK have been increasing for several years.
- Non-peer-reviewed work has claimed a strong relationship between Delayed Transfers of Care (DTOCs) and patients waiting for long periods in EDs.
- The peer-reviewed evidence that exists currently is limited but suggests that the relationship is weaker than expected.

**What this study adds**

- A critique of the current analyses of association between DTOCs and ED waiting times.
- A demonstration of why the time series structure of data are important.
- Understanding that DTOCs are important to patients and their care, but further research on the impact of improving DTOCs on ED waiting times is required.

## Introduction

There has been increasing pressure on Emergency Department (ED) services worldwide in recent years, with much attention focused in the United Kingdom (UK). Adherence to the UK target of 95% of patients attending EDs being seen, treated and discharged within 4 hours has reduced dramatically in recent years.^1^

One of the suggested causes of the inability of UK EDs to meet the 4-hour target has been the increase in Delayed Transfers of Care (DTOCs).^2 3^ NHS England defines DTOCs as occurring “when an (inpatient) is ready to depart from care and is still occupying a bed”.^4^ An inpatient is ready to depart when the following are true:

- A clinical decision has been made that a patient is ready for transfer.
- A multi-disciplinary team decision has been made that a patient is ready for transfer.
- The patient is safe to discharge/transfer.

DTOCs must be routinely reported by hospital trusts. It intuitively makes sense that DTOCs could affect ED performance. If an inpatient is not discharged from an inpatient area of a hospital the bed cannot be made available for new patient admissions from the ED. Many DTOCs present within the hospital at one time could limit bed availability for emergency patients who require admission throughout a given day.

An analysis published in 2016^2^ reported there was “an observed positive association between the numbers of emergency department patients waiting more than 4 hours to be either admitted into hospital, transferred out of the department, or discharged home and the number of patients waiting to vacate a bed elsewhere in the hospital”. The Nuffield Trust^3^ referenced this study stating: “There is a strong link between DTOCs and patients waiting for extended periods in the (ED) department.” The analysis was based on open NHS data from 2010 to 2016 in which the author used linear regression and found an association between DTOCs and breaches. However, an unrecognised limitation of the study was that it did not follow standard statistical inference procedures for data that are repeatedly measured over equally spaced time intervals. Furthermore the data utilised in the study was analysed at a national level as opposed to individual hospital level. Such aggregated data limits the type of observational study that can be performed to an *ecological study*. Ecological studies are generally thought of as hypothesis-generating only.^5^ This is in contrast to other observational study types, such as *case-control* or *cohort studies,* which conduct analysis at individual level and, if appropriate, can provide a stronger level of evidence. In contrast to findings of the 2016 analysis, more recent work using the same NHS England open data (for the period 2012–2016) demonstrated, in a series of cross-sectional studies, that there was only limited evidence of an association between these variables.^6^

### Importance of considering time series structure of data

Measurements repeatedly taken in time are referred to as a *time series* in statistical terminology. Formally we define a time series as a sequence of data points of a common metric with temporal ordering: for example, the number of copies of a national newspaper sold per day in 1981 or the number of DTOCs per month between September 2010 and September 2012. Estimating an association between two or more time series variables is a special case of time series analysis. If, for example, trends within the data are ignored, analyses can lead to spurious results that over- or understate an association.

To use standard analysis methods, such as multivariate regression, when dealing with time series the data must be *stationary*, that is, the mean, variance and autocorrelation of the data must be constant through time. When data display trends, the technical description is *non-stationary*. Thus, appropriate practice in a multiple time series analysis is to first assess if a trend is present in each time series and apply procedures to remove these. Autocorrelation refers to the concept that a data point within a series is often highly correlated with prior historical observations of the same variable. In ordinary least squares regression analysis, autocorrelation also affects the model error series (differences between predicted and actual outcomes) which, in turn, leads to standard errors of the coefficients which are too small. Interpreting these can therefore lead to spurious conclusions. Time series methods provide statistical treatments of the data to model this autocorrelation and adjust results.

In many cases in real life it is unusual for the mean, variance and autocorrelation to be constant over time. For example, consider the mean number of patients seen each day in the ED over the course of the year, or their waiting time. Spurious results can occur in analyses that do not take into account the time series nature of the data.^7 8^ Trends can be removed by *differencing* or by *subtracting models* that have been fitted to the time series. The full breadth of approaches for detrending is beyond the scope of this article, but interested readers are referred to Cowpertwait et al^9^ or any other statistical text that deal with time series.

The aim of this study was to demonstrate how two artificial datasets with no logical correlation can appear correlated if they have a similar upward trend and perform a reanalysis of the observational study reported in^2 3^ making use of standard techniques from the branch of statistics known as *time series analysis*. Our conclusions discuss the potential pitfalls when interpreting ecological hypothesis-generating studies and propose the next steps in a more comprehensive analysis of DTOCs and ED crowding.

## Method

### Study structure

We conduct two analyses in this study. Our analyses here focus on the consequences of coincident trends within multiple time series and their impact on the association between ED breaches and DTOCs. Analysis 1 uses artificially-generated (synthetic) data to demonstrate that analyses which do not account for the time series nature of data, can lead to incorrect conclusions about correlation. Synthetic data refers to data which is created by an algorithm artificially, as opposed to being collected in the ‘real world’. Analysis 2 applies the same analytic method to actual DTOCs and ED waiting-time data to demonstrate that the same phenomena can occur in ‘real’ data. We then conduct a correlational analysis using appropriate methods to account for the time series nature of the data.

### Analysis 1 – synthetic time series analysis

Two sets of time series data were generated by randomly sampling a set of numbers from a normal distribution and placing them in a sequence. We named these Time Series A and Time Series B. There was no correlation displayed between the two time series at this stage. An upward linear trend was applied separately to each time series so that each set of data increased over time. This was done by adding a value to each number in the sequence, which increased from the beginning to the end of the sequence. Each data set was therefore non-stationary as the mean increased over time.

We plotted each time series separately and combined them into a scatter plot. We conducted a univariate linear regression of Time Series A against Time Series B. An explanation of the code used to generate these time series, and conduct the analysis, is available in (online supplementary material appendix A).

10.1136/emermed-2018-207917.supp1 Supplementary data

### Analysis 2 – reproduction of previous study and reanalysis with detrending

This analysis used openly-published NHS England statistics on ED waiting times and DTOCs between August 2010 and April 2016.^10^ This was the same time period used in the previous study.^2^ The analysis was conducted at national level with monthly intervals using the NHS England data. The variables investigated in this study were: ‘number of breaches’ of the 4-hour waiting time target (referred to as breaches herein), and ‘number of delayed transfers of care’ (DTOCs). We used the same analytical method as in analysis 1: we plotted each time series separately and combined them to create a scatter plot. We conducted a univariate linear regression of the number of DTOCs against breaches as was done in the previous study.^2^ As in the previous study we report the coefficient of determination (R^2^) which gives an indication of the proportion of the variance in breaches that is predictable from DTOCs.

We then compared the results of this regression analysis with those of a method using standard time series approaches to account for the time series nature of the data. Viewing the data as a time series, we tested for stationarity using an augmented Dickey-Fuller test and examined autocorrelation visually using a plot of the autocorrelation function.^9^ We then detrended both time series by fitting and subtracting (polynomial) models to the data. We conducted the same univariate linear regression described above and reported the R^2^. We provided further analysis by completing Spearman’s correlation calculations on these detrended time series. The complete working can be seen in online supplementary appendix B.

10.1136/emermed-2018-207917.supp2 Supplementary data

### Tools and reproducibility

The analysis conducted in this work was completed using the python language (V.3.6.0; www.python.org). All computational code and data are available in supplementary materials to fully reproduce the analysis.

## Results

### Analysis 1 – synthetic time series analysis

**Figure 1:**
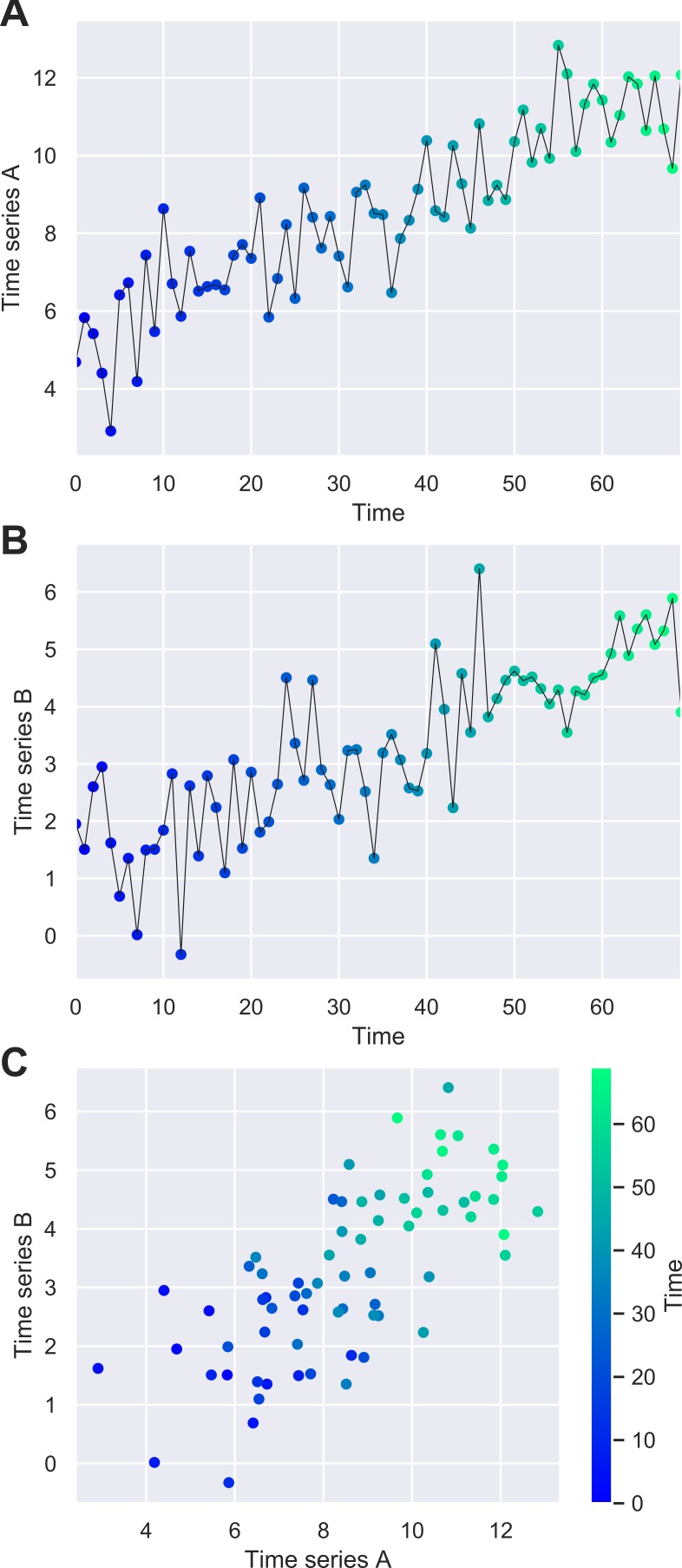
Line plot showing synthetic data of (A) Time Series A and (B) Time Series B; (C) scatter plot of the same points which demonstrate a strong positive correlation (R^2^=0.51).

Figure 1 illustrates the artificial data for: (A) Time Series A and (B) Time Series B. Figure 1C illustrates a scatter plot of the same points where the temporal ordering is not considered. Figure 1C indicates a strong positive correlation which was confirmed by the linear regression analysis (R^2^=0.51). The shading of the scatter plot shows the temporal ordering of the points. The starting points of each time series are observed in the bottom left corner, while the ending points of each series are observed in the top right corner. This illustrates that the upward trends lead to the correlation observed between Time Series A and Time Series B which, before the upward trend was added, were otherwise uncorrelated.

### Analysis 2 – use of real data to reproduce the previous study

**Figure 2:**
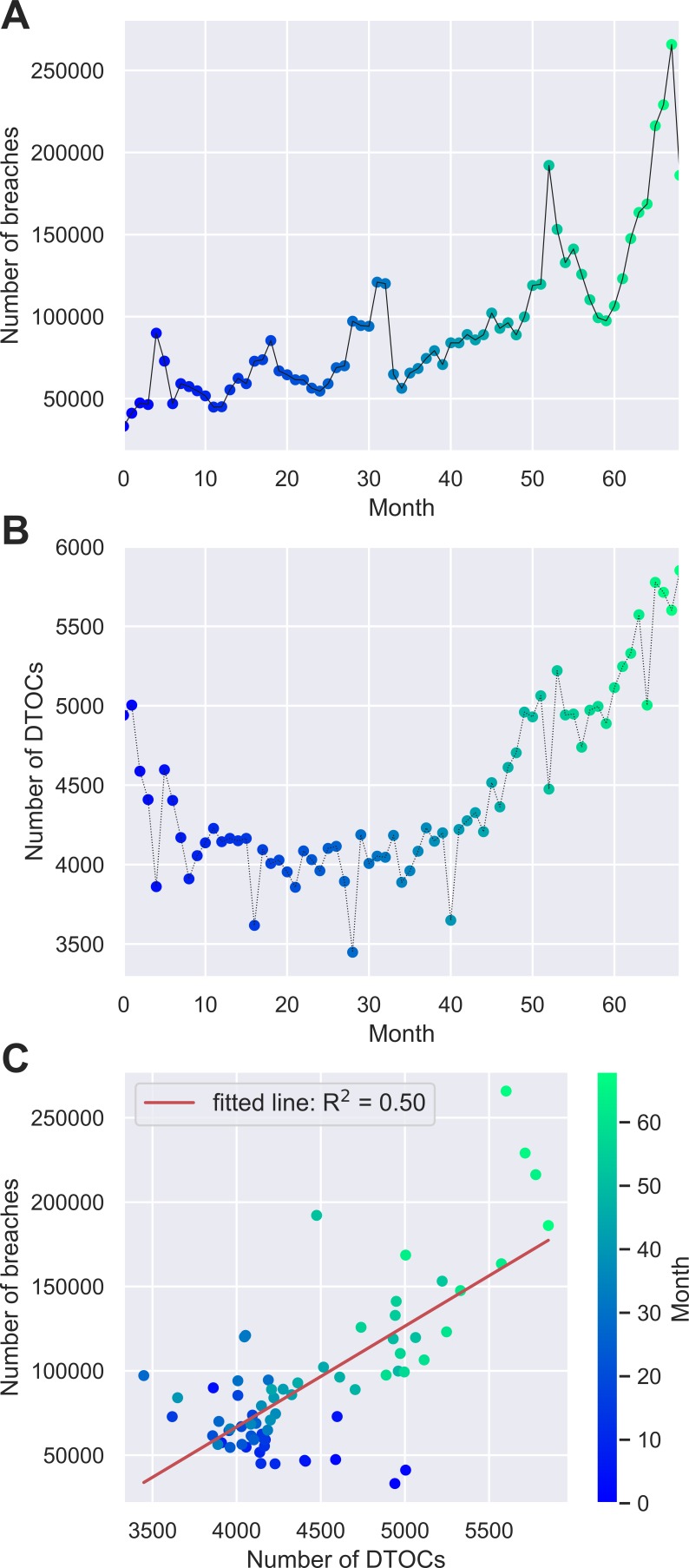
Line plot showing the time series of (A) number of breaches and (B) number of DTOCs; (C) a reproduction of the scatter plot previously published.^2^

Figure 2 illustrates the time series of: (A) number of breaches and (B) number of DTOCs. Figure 2C shows a reproduction of the analysis in the previous study,^2^ showing the scatter plot with high coefficient of determination value (R^2^=0.50).

### Analysis 2 – reanalysis of previous study with detrending

**Figure 3:**
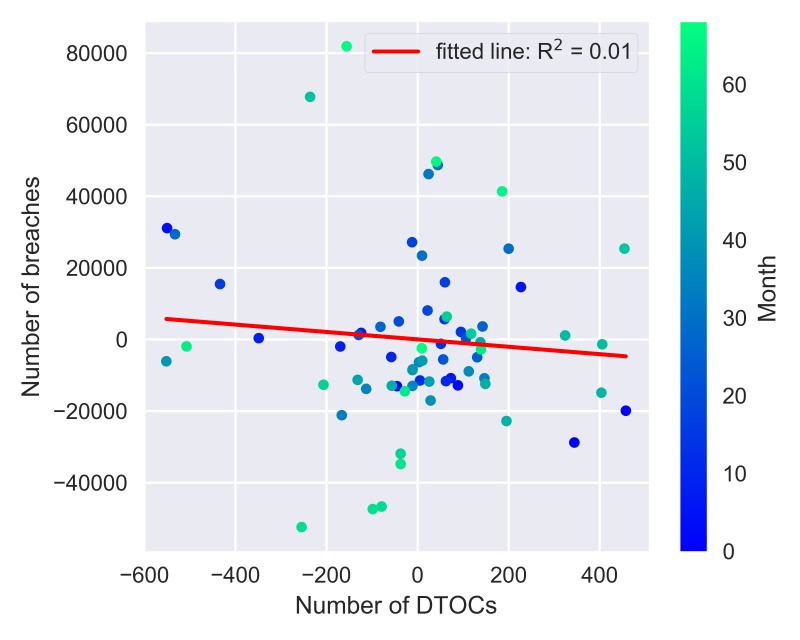
Scatter plot and analysis on the detrended NHS England data.

Augmented Dickey-Fuller tests yielded P-values=0.51 and 0.99 respectively for the breaches and DTOC time series, demonstrating no statistical evidence to support trend-stationarity in either time series. This supports the visual evidence in figure 2A and B of an increasing mean value observed over time. The autocorrelation function indicated considerable autocorrelation was present (see online supplementary appendix B). Figure 3 illustrates the same analysis conducted above but applied to the detrended time series (R^2^=0.01). The R^2^ value obtained before detrending is much higher than for the detrended time series.

Table 1 reports the correlation coefficient and associated P-values for both the original data and the detrended time series. The coefficient values for the detrended data are lower than those found in the original unadjusted data.

**Table 1:** Correlation coefficient values for the original and the detrended data

	Spearman’s correlation coefficient*	P-value
Original data	0.54	<0.005
Detrended	0.02	0.88

*Original data exhibited signs of non-normality, hence Spearman’s correlation coefficient is presented.

## Discussion

Given the nature of patient flow through a hospital, it is plausible to hypothesise that DTOCs are causally associated with breaches of the ED waiting time target. Our reanalysis demonstrates that there are several statistical and conceptual issues that mean the current quantitative evidence to support such a statement has considerable uncertainty.

### Interpretation of this study

Our study demonstrates that the substantive correlation reported in^2 3^ is unreliable and indicates only that each series in the analysis has a long-term upward trend. The current evidence of association between DTOCs and ED breaches is not of high quality and more research is needed. We make no claims that there is no relationship between the two variables, but instead that there is insufficient quantitative evidence to strongly support the proposition.

In order to estimate the association between two or more time series in the NHS England data reported in,^2 3^ time series analysis methods are required. We found strong evidence of longitudinal trend and autocorrelation of model errors in our reanalysis of^2^ and hence the simple linear regression methodology used in the original analysis is unsuitable. The result reported in^2 3^ is equivalent to the analysis of our synthetic data that contain a coincident trend, but no ‘real’ correlation. Our reanalysis of the original study, accounting for the trends over time, found no evidence of an association between numbers of breaches and numbers of DTOCs. Other ED studies involving repeated measurements made in time have found similar differences in their conclusions when comparing simple statistical analyses with those that account for the time series nature of their data.^7 8^

### A causal effect or spurious correlation?

Spurious correlations refer to relationships between variables that demonstrate correlation but in fact have no meaningful (causal) association. These can occur due to chance or to the presence of a third hidden variable.

Examples due to chance occur because if enough comparisons are made between different data sets, eventually we will find two sets of data which display a high correlation. Examples include randomly-generated series of data^9^ and the association of ‘number of people who drowned falling into swimming pools’ in the USA with ‘films Nicholas Cage appeared in’ 1999 to 2009.^11^ One other example of this can be found in the NHS England data of the original study. If we were to conduct the same analysis for only months 0–30 (August 2010 – March 2013) we would find a negative correlation. This can be seen when considering the blue points in figure 2C and is due to the initial downward trend in the DTOC data. Following the same interpretation as the original study we would hence conclude that increasing number of breaches nationally was related to the *reduction* in the number of DTOCs nationally.

Confounding or ‘hidden’ variables, not included in the analysis, can also explain an association found between two variables. It is common in ecological study types to include other variables in the analysis which helps to exclude the possibility of these alternate explanations of the results.^5^ For example, in this study it is plausible that increases in the number of patient visits to EDs or increases in the average inpatient bed occupancy may be associated with the increases in numbers of breaches nationally. A rigorous analysis of the relationship would adjust for these parameters.

### What does a lack of 'strong association' mean?

It indicates that there is little short-term association between fluctuations in numbers of DTOCs and breaches nationally: over the period studied, months with higher numbers of DTOCs nationally did not correspond with higher numbers of breaches nationally. There are some reports that suggest DTOCs are potentially underreported,^3^ which is likely to vary between trusts. It is plausible if a correlation does exist at national level, after detrending, it is masked due to this additional uncertainty in these measurements.

As this is an ecological study finding no association at national level does not exclude the possibility that a relationship exists when analysing data at individual hospital level. A recent hospital-level study has shown that DTOCs are associated with inpatient bed occupancy, and that occupancy is related to breaches.^6^ This may mean that DTOCs are only one aspect of a wider issue that trusts are facing around patient flow. The study found the link between patient flow and breaches is not particularly strong, although a weakness of the analysis was that it assumed linear relationships.

A lack of strong association implies limited support for the hypothesis that ED waiting times are associated with DTOCs, but it does not mean DTOCs are unimportant. There are many reasons to tackle DTOCs, including patient experience and aspects of patient safety.

### Uncertainty in the evidence base

Over-interpretation of results without consideration of limitations of the statistical analysis, and references to non-peer reviewed work, can lead to beliefs in associations which do not exist or, at the very least, currently have limited evidence to support them. As researchers and decision makers within the the NHS we must be careful not to over-interpret evidence that supports our intuitions. Otherwise it is likely that we enforce changes to services which do not provide patients with better care, while adding to the ‘change fatigue’ that frontline staff already face.

### What next to understand patient flow?

Development of this ecological study, to include possible confounding variables, could further this discussion. Cohort studies using open data available at hospital level^12^ could provide further insight into how changes in patient flow affects individual hospitals in different ways. Such studies must involve appropriate statistical methodologies which can account for the time series nature of data as well as differences in individual hospital effects. More peer-reviewed studies of patient flow within hospital trusts are also required: these would have the advantage of understanding the local context of data collection and measurement error, which potentially weaken associations. One such peer-reviewed study exists although it does not acknowledge the issues we have presented when analysing time series data.^13^

### Limitations

Both this study and the original analysis^2^ are examples of ecological studies as measurements are grouped from individual hospital-level data but analysed together in a group. Ecological studies are commonly used to generate hypotheses, which may be worth investigating using more rigorous epidemiological methodologies,^5^ but are known to have severely limited causal inference.^14^ One important subtlety, which was not addressed in the original study, is that we can only comment on the association at a national level. In this study we are *unable* to assume that any association that exists between DTOCs and numbers of breaches at the group level (national level) also exists at individual level (hospital level): any association could be stronger, weaker or not even present at the individual level. To make conclusions at individual level in ecological studies is known as an *ecological fallacy*.

The simple analysis we present in this study is limited in its approach to detrending. A full analysis should consider other methods of detrending and statistical methods which can account for time series’ data structure.

## Conclusion

We have highlighted that the strong association previously reported between DTOCs and breaches may be due only to the long-term trend and violates the statistical assumptions of the method being used. Furthermore, both our study and the original are severely limited in their causal inference because they are ecological. Our study does *not* indicate that there is no relationship between breaches and DTOCs, it highlights that high quality and statistically robust evidence for this relationship does not currently exist.
