# Supplementary material for: The impact of delayed transfers of care on emergency departments: common sense arguments, evidence and confounding
Source: Emerg Med J. 2019 Nov 25;37(2):95–101. doi: 10.1136/emermed-2018-207917 (PMC7027036; doi:10.1136/emermed-2018-207917)
Supplement: Supplementary data [file emermed-2018-207917supp001.pdf]

Appendix A

October 3, 2019

This jupyter notebook provides an example of how two series of values that have very low correlation scores (and have no reason to be correlated), can have seemingly high measures of correlation if there is an arbitary trend occuring over the series.

Any set of observations of values, that are measured at discrete intervals in time can be reffered to as a time series. We will use the term time series in this appendix.

Import libraries

```
In [1]: import numpy as np
        import pandas as pd
        from numpy.random import random_sample
        import scipy
        from scipy import stats

        import matplotlib.pyplot as plt
        import seaborn as sns

        %matplotlib inline
        sns.set()
```

1 Correlations between two random lists of measurements

creating two sets of random values: a & b. a & b can be thought of as individual time series.

```
In [2]: i = 70 # number of points to create
        np.random.seed(6) # set seed for random generation: so produces the same random sample for
        rand = pd.DataFrame({'a': np.random.randn(i),'b':np.random.randn(i)})
        rand['a'] = rand['a'] + 5
        rand['b'] = rand['b'] + 1
```

Showing the first 5 points in each timeseries:

```
In [3]: rand.head()
```

```
Out[3]:          a         b
        0  4.688216  1.951201
        1  5.729004  1.441897
```


Keogh B, Monks T. *Emerg Med J* 2020; 37:95–101. doi: 10.1136/emermed-2018-207917

```
2  5.217821  2.469017
3  4.100908  2.749516
4  2.513219  1.353531
```

Plot points in each time series

```
In [4]: fig, ax = plt.subplots();
        rand.plot(figsize=(16,4),ax=ax);
        ax.legend(frameon=True);
        ax.set_ylabel('Value');
        ax.set_xlabel('Time point');
```

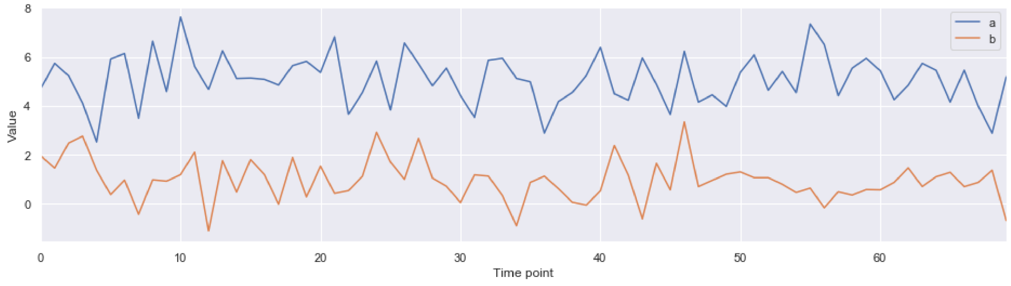

Calculating the pearson correlation between the values in each time series: we find there is low/no correlation (-0.001) between a & b.

```
In [5]: # calc correlation matrix
        rand.corr('pearson').round(3)

Out[5]:        a      b
        a  1.000 -0.001
        b -0.001  1.000
```

2 Adding an arbitrary linear upward trend to each time series…

```
In [6]: rand['a_with_trend'] = rand.a + rand.index/10

In [7]: rand['b_with_trend'] = rand.b + rand.index/15

In [8]: fig, ax = plt.subplots();
        rand[['a_with_trend','b_with_trend']].plot(ax=ax);
        ax.legend(frameon=True);
        ax.set_ylabel('Value');
        ax.set_xlabel('Time point');
```


Keogh B, Monks T. *Emerg Med J* 2020; 37:95–101. doi: 10.1136/emermed-2018-207917

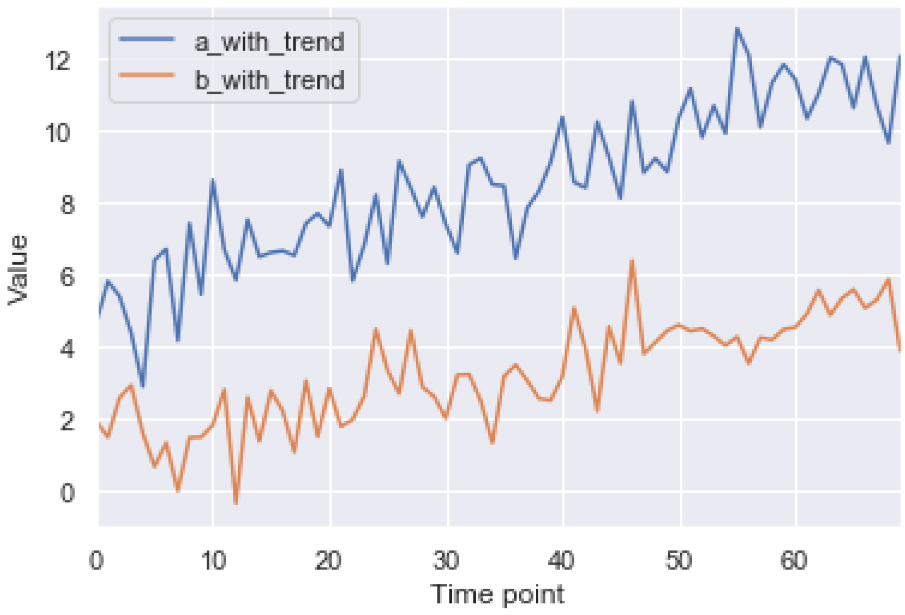


```
In [9]: # calc correlation matrix
        rand.corr('pearson').round(2)

Out[9]:                  a     b  a_with_trend  b_with_trend
        a             1.00 -0.00          0.39         -0.08
        b            -0.00  1.00         -0.19          0.40
        a_with_trend  0.39 -0.19          1.00          0.72
        b_with_trend -0.08  0.40          0.72          1.00
```

We observe the spearman rank correlation coefficient between each list with added trend (a_with_trend & b_with_trend) is much greater (0.72).

3 Removing the trend in the time series

We fit a polynomial to each series: $y = mx + c$. We remove the trend by subtracting the polynomial.

```
In [10]: def func_dtoc(x, m, c):
             return (m * x)  + c

In [11]: def detrend_using_func(dta,column,func,ylabel,color='b'):

             #### fit func
             xdata = dta.index
             ydata = dta[column]
             popt, pcov = scipy.optimize.curve_fit(func, xdata, ydata)
```

```
    #### plot fig
    fig = plt.figure() # Create matplotlib figure

    ax = fig.add_subplot(111) # Create matplotlib axes
    dta[column].plot(color=color, ax=ax, label=ylabel)#, width=width)#, position=0)
    plt.plot(xdata, func(xdata, *popt), color+ '--',
             label='fit:m=%5.3f, c=%5.3f' % tuple(popt)) #

    ax.set_ylabel(ylabel)
    ax.legend(frameon=True)
    plt.show()

    #### make new column with detrended data
    dta.loc[dta.index,column[0]+'_detrend'] = dta[column] - func(dta[column].index,popt[0],pop

    return dta
```

```
In [12]: rand = detrend_using_func(rand,'a_with_trend',func_dtoc,'a_with_trend')
```

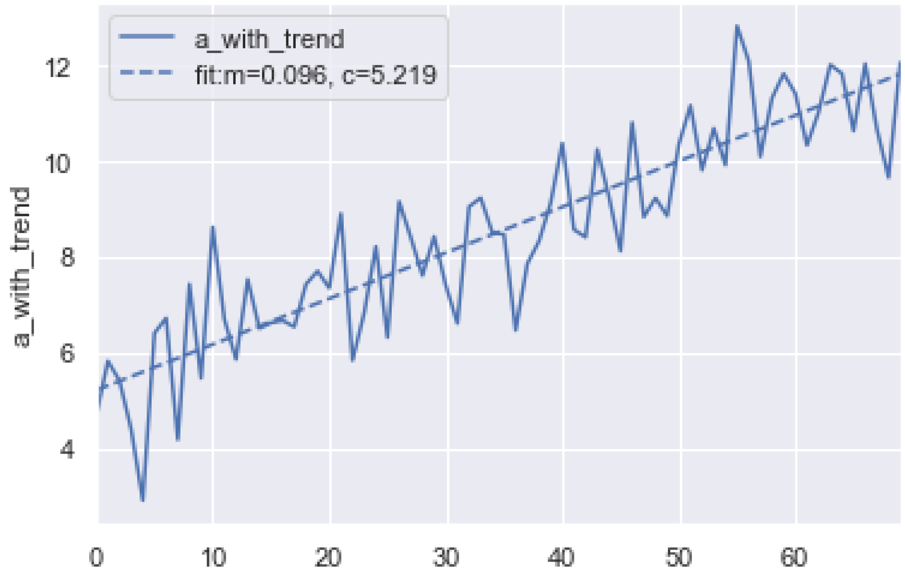


```
In [13]: rand = detrend_using_func(rand,'b_with_trend',func_dtoc,'b_with_trend','g')
```


Keogh B, Monks T. *Emerg Med J* 2020; 37:95–101. doi: 10.1136/emermed-2018-207917

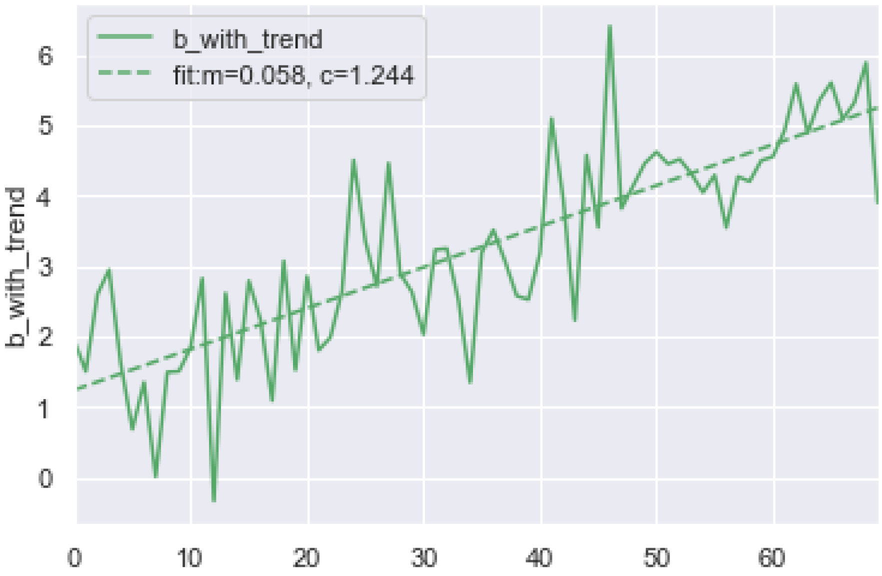


```
In [14]: # calc correlation matrix
         rand.corr('pearson').round(2)
```

Out[14]:

	a	b	a_with_trend	b_with_trend	a_detrend	b_detrend
a	1.00	-0.00	0.39	-0.08	1.00	-0.02
b	-0.00	1.00	-0.19	0.40	-0.02	0.98
a_with_trend	0.39	-0.19	1.00	0.72	0.47	-0.01
b_with_trend	-0.08	0.40	0.72	1.00	-0.01	0.58
a_detrend	1.00	-0.02	0.47	-0.01	1.00	-0.02
b_detrend	-0.02	0.98	-0.01	0.58	-0.02	1.00

The pearson correlation value between the detrended time series (a_detrend & b_detrend) is found to be very small: -0.02. The value is closer to the original correlation value calculated between the time series (a & b): -0.001.

4 Conclusion

We conclude it is possible that two uncorrelated time series can display pearson correlation values much higher than would otherwise be present with the presence of long term trends.

5 Plot & calculations for paper

Figure 1

Keogh B, Monks T. *Emerg Med J* 2020; 37:95–101. doi: 10.1136/emermed-2018-207917

```
In [15]: #### prep figure and data
         fig,ax = plt.subplots(3,1,figsize=(5,11));
         df = rand.copy()
         df['Time'] = df.index;
         df['Time series A'] = df['a_with_trend'];
         df['Time series B'] = df['b_with_trend'];

         #### line plots
         df[['Time series A']].plot(ax=ax[0],color='k', linewidth=0.5, legend=False);
         df[['Time series B']].plot(ax=ax[1],color='k', linewidth=0.5, legend=False);

         #### scatter plots
         cmap='winter'
         df[['Time','Time series A']].plot('Time','Time series A',kind='scatter',ax=ax[0],c='Time',colo
         df[['Time','Time series B']].plot('Time','Time series B',kind='scatter',ax=ax[1],c='Time',colo
         df[['Time series A','Time series B','Time']].plot('Time series A','Time series B',kind='scatte

         #### plot tidying
         fig.tight_layout(pad=1)

         plt.text(0.9, 25.1, 'a', fontsize=18);
         plt.text(0.9, 15.9, 'b', fontsize=18);
         plt.text(1, 6.7, 'c', fontsize=18);

         # fig.savefig('Figure1',dpi=800)
```


Keogh B, Monks T. *Emerg Med J* 2020; 37:95–101. doi: 10.1136/emermed-2018-207917

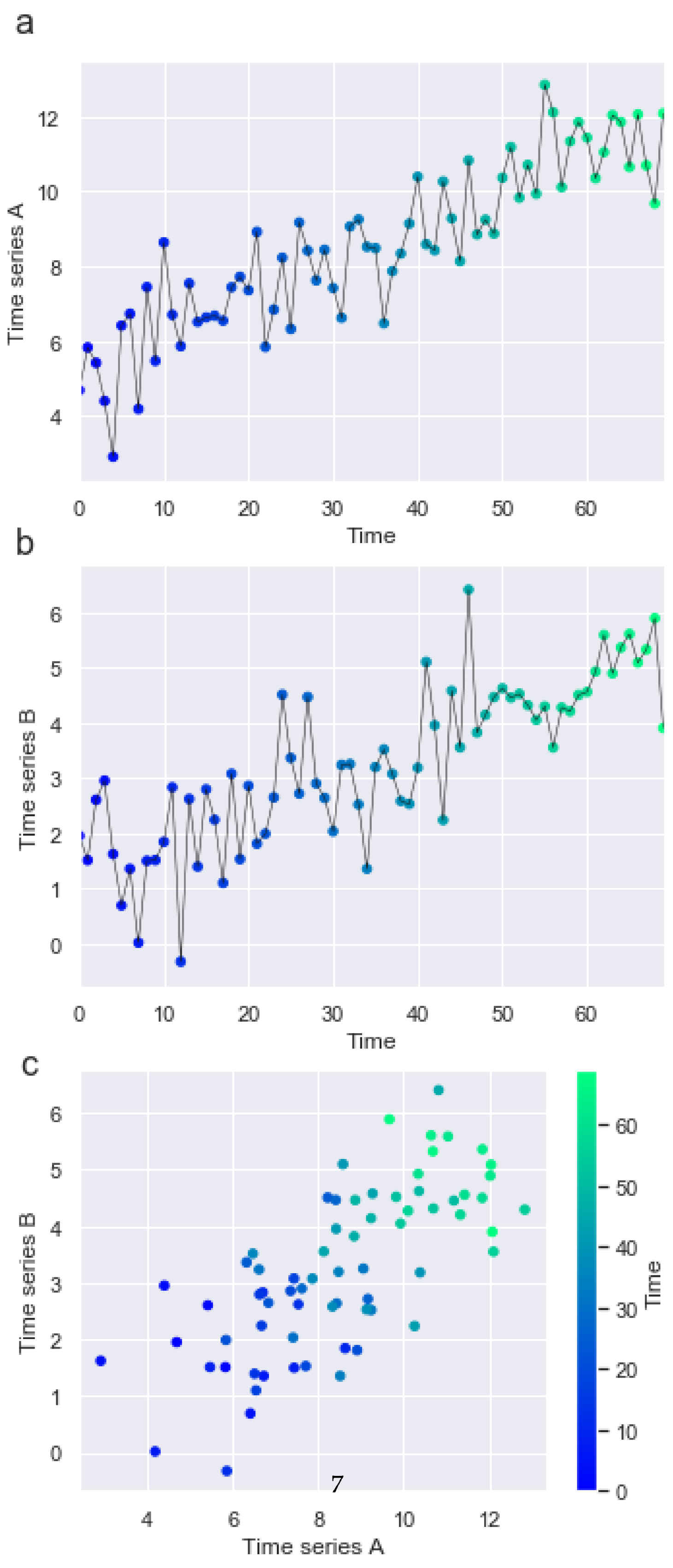
a
Time series A
Time
b
Time series B
Time
c
Time series B
Time series A
Time

Ordinary least squares regression:

```
In [16]: slope, intercept, r_value, p_value, std_err = stats.linregress(df['b'],df['a'])

         print('R^2 value of original random data is: ', (r_value**2).round(4))

R^2 value of original random data is:  0.0
```

```
In [17]: slope, intercept, r_value, p_value, std_err = stats.linregress(df['Time series B'],df['Time se

         print('R^2 value of data with added linear trend is: ', (r_value**2).round(2))

R^2 value of data with added linear trend is:  0.51
```

```
In [18]: slope, intercept, r_value, p_value, std_err = stats.linregress(df['b_detrend'],df['a_detrend']

         print('R^2 value of de-trended data is: ', (r_value**2).round(4))

R^2 value of de-trended data is:  0.0003
```


Keogh B, Monks T. *Emerg Med J* 2020; 37:95–101. doi: 10.1136/emermed-2018-207917
