# Supplementary material for: The impact of delayed transfers of care on emergency departments: common sense arguments, evidence and confounding
Source: Emerg Med J. 2019 Nov 25;37(2):95–101. doi: 10.1136/emermed-2018-207917 (PMC7027036; doi:10.1136/emermed-2018-207917)
Supplement: Supplementary data [file emermed-2018-207917supp002.pdf]

Appendix B

October 3, 2019

This jupyter notebook contains analysis of the counts of 'ED four-hour target breaches' and 'Delayed Transfers Of Care' at English Trusts August 2010 -> April 2016. It contains all the complete analysis steps used in the paper.

import libraries

```
In [1]: import numpy as np
        import pandas as pd
        import scipy
        from scipy import stats
        import statsmodels.api as sm

        import matplotlib.pyplot as plt
        import seaborn as sns

        sns.set()
        %matplotlib inline
```

1 Load data

```
In [2]: df = pd.read_csv('NHSE_data.csv')
        df.shape

Out[2]: (80, 10)

In [3]: df.head()

Out[3]:       year      month  total_attendances  total_attendances_t1  \
        0  2010-11     August        1719197.000           1138652.000
        1  2010-11  September        1715117.000           1150728.000
        2  2010-11    October        1753934.000           1163143.000
        3  2010-11   November        1604591.000           1111294.571
        4  2010-11   December        1647823.857           1159203.857

           total_breaches  95%_targ  total_admissions  dtoc_a  dtoc_na  total_dtocs
        0     33184.00000  0.980698       425702.0000    2559     2381         4940
        1     41151.00000  0.976007       424900.0000    2647     2357         5004
```


Keogh B, Monks T. *Emerg Med J* 2020; 37:95–101. doi: 10.1136/emermed-2018-207917

```
2     47414.00000  0.972967       436215.0000    2513     2075          4588
3     46436.42857  0.971060       429099.0000    2352     2057          4409
4     89917.28571  0.945433       452728.7143    1995     1866          3861
```

make flag for data used in previous analysis

```
In [4]: df['flag_previous'] = 0

        df.loc[0:68,'flag_previous'] = 1
```

remove most recent data to reproduce previous analysis.

```
In [5]: df = df[df.flag_previous == 1]

In [6]: df = df[['total_breaches','total_dtocs']] # select only variables of interest
```

2 Data briefing reproduction

A reproduction of the analysis in BMJ data briefing by Appleby: https://www.bmj.com/content/353/bmj.i3585

```
In [7]: # get coeffs of linear fit
        slope, intercept, r_value, p_value, std_err = stats.linregress(df['total_dtocs'],df['total_brea

In [8]: fig, ax = plt.subplots(figsize=(6,5))
        y = df['total_breaches']
        x = df['total_dtocs']
        plt.plot(x,y,'o', label='monthly data')
        plt.plot(df['total_dtocs'], intercept + slope*x, 'r', label='fitted line: R$^2$ = {0:.2f}'.form
        plt.legend(frameon=True,fontsize='large')
        ax.set_xlabel('number of DTOCs')
        ax.set_ylabel('number of breaches')
        plt.show()
```

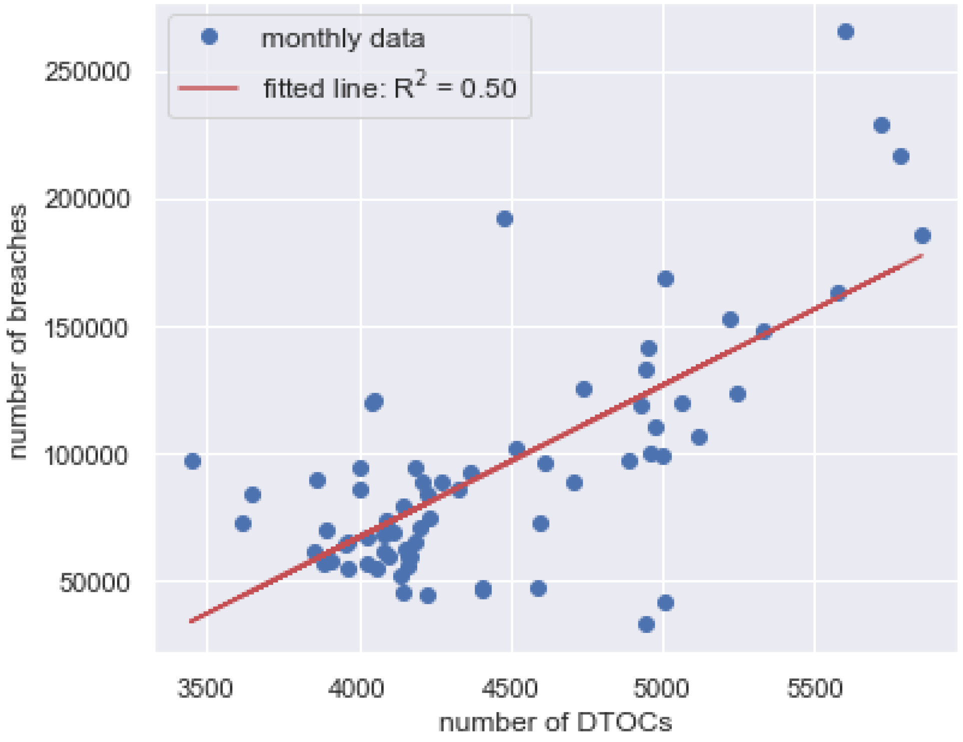

We find similar R2 value that was found in the previous analysis:

```
In [9]: (r_value**2).round(3)
```

```
Out[9]: 0.504
```

distribution of data Check distribution of each variable.

```
In [10]: df.hist();
```

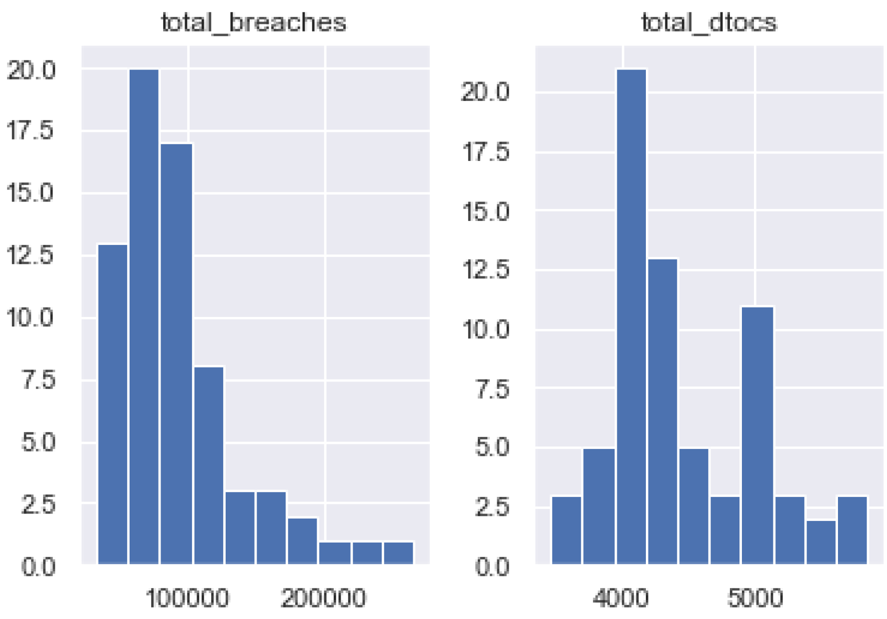

correlation coefficient Data appears to not be normally distributed. Hence, we should avoid using Pearson correlation coefficient. Calculate correlation values:

```
In [11]: from scipy.stats.stats import pearsonr

In [12]: corr, p = pearsonr(df['total_dtocs'],df['total_breaches'])

In [13]: print('Pearson')
         print('Coeficient: ', corr)
         print('P-value' , p)

Pearson
Coeficient:  0.710060292485
P-value 8.37892018508e-12


In [14]: from scipy.stats.stats import spearmanr

In [15]: corr, p = spearmanr(df['total_dtocs'],df['total_breaches'])

In [16]: print('Spearman')
         print('Coeficient: ', corr)
         print('P-value' , p)

Spearman
Coeficient:  0.541634696551
P-value 1.53991353597e-06
```

3 Investigate timeseries properties

Stationarity One method to asses if timeseries is stationary is to use an augmented dickey-fuller test. This tests to see if a unit root is present within the data - which would indicate non-stationarity.

```
In [17]: from statsmodels.tsa.stattools import adfuller
```

```
In [18]: def check_stationarity(series):
             result = adfuller(series)
             print('ADF Statistic: %f' % result[0])
             print('p-value: %f' % result[1])
             for key, value in result[4].items():
                 print('\t%s: %.3f' % (key, value))
```

```
In [19]: check_stationarity(df['total_breaches'])
```

```
ADF Statistic: -1.555714
p-value: 0.505793
        1%: -3.530
        5%: -2.905
        10%: -2.590
```

```
In [20]: check_stationarity(df['total_dtocs'])
```

```
ADF Statistic: 1.641969
p-value: 0.997978
        1%: -3.551
        5%: -2.914
        10%: -2.595
```

The p-value » 0.05 indicates that there is a unit root present in both of the timeseries. This implies each variable is non-stationary.

Assess level of autocorrelation using ACF

```
In [21]: fig = sm.graphics.tsa.plot_acf(df['total_breaches'],lags=18)
```


Keogh B, Monks T. *Emerg Med J* 2020; 37:95–101. doi: 10.1136/emermed-2018-207917

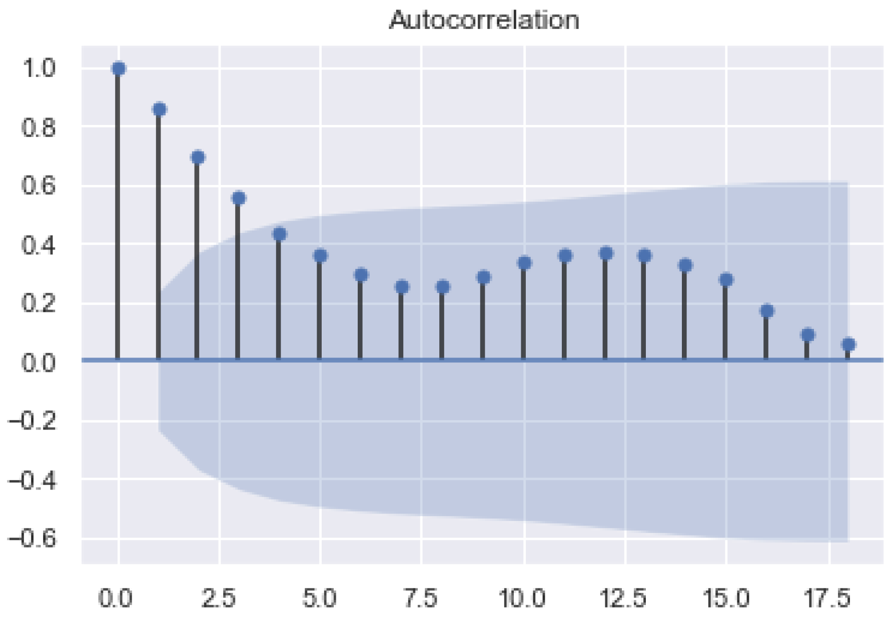


```
In [22]: fig = sm.graphics.tsa.plot_acf(df['total_dtocs'],lags=18)
```

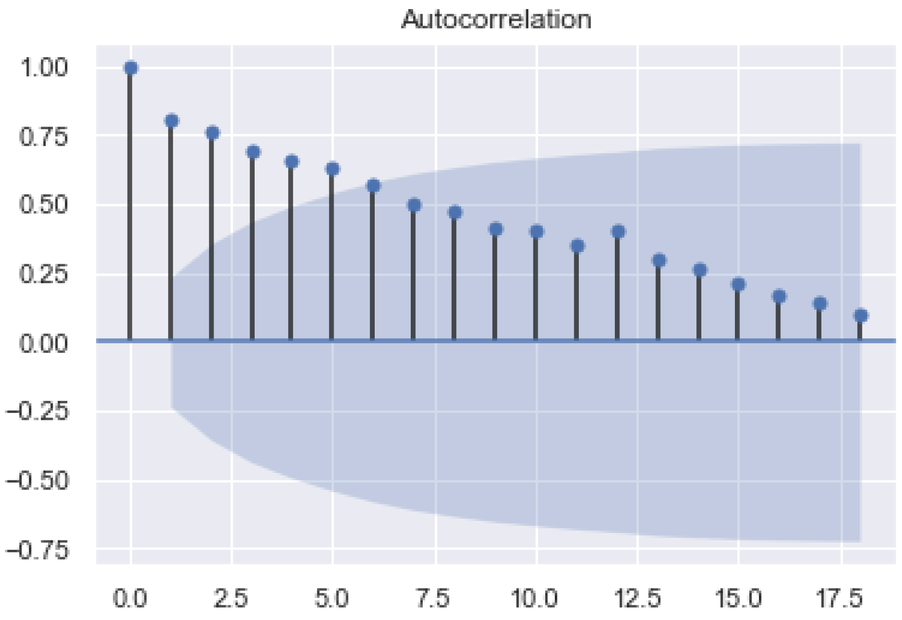

High autocorrelation values for many lags in both timeseries demontrate that each time point is highly dependent on the previous ones. This may indicate an increasing growth over time; one cause of a timeseries being non-stationary. This is very clear upon plotting the time-series below.

In [23]:
```
fig = plt.figure() # Create matplotlib figure

ax = fig.add_subplot(111) # Create matplotlib axes
ax2 = ax.twinx() # Create another axes that shares the same x-axis as ax.

df.total_breaches.plot(color='red', ax=ax)#, width=width)#, position=1)
df.total_dtocs.plot(color='blue', ax=ax2)

ax.set_ylabel('Number of breaches')
ax2.set_ylabel('Number of dtocs')
ax2.grid(b=False)

#sort legend
lns = ax.get_lines()+ax2.get_lines()
ax.legend(lns,['breaches','DTOCs'],frameon=True)

plt.show()
```

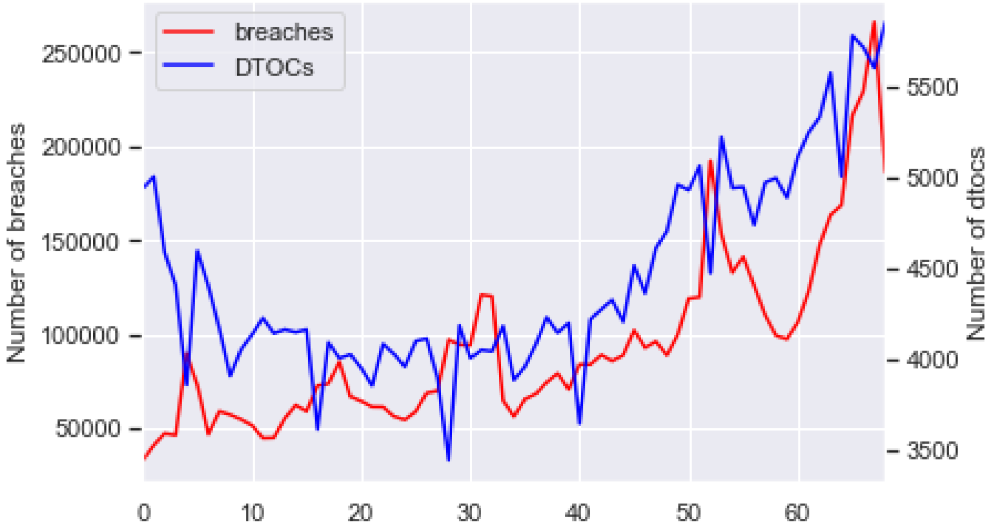

4 Detrend using fitted polynomial

One method to detrend the time-series is by fitting polynomial equations and subtracting these from the time-series.

```
In [24]: def func_dtoc(x, a, b, c):
             return (a * x) + (b * x**2) + c

In [25]: def detrend_using_func(dta,column,func,ylabel,color='b'):

             #### fit func
             xdata = dta.index
             ydata = dta[column]
             popt, pcov = scipy.optimize.curve_fit(func, xdata, ydata)

             #### plot fig
             fig = plt.figure() # Create matplotlib figure

             ax = fig.add_subplot(111) # Create matplotlib axes
             dta[column].plot(color=color, ax=ax, label=ylabel)#, width=width)#, position=0)
             plt.plot(xdata, func(xdata, *popt), color+ '--',
                      label='fit:a=%5.3f,  b=%5.3f, c=%5.3f' % tuple(popt)) #

             ax.set_ylabel(ylabel)
             ax.legend(frameon=True)
             plt.show()

             #### make new column with detrended data
             dta.loc[dta.index,column+'_detrend'] = dta[column] - func(dta[column].index,popt[0],popt[1

             return dta

In [26]: df = detrend_using_func(df,'total_dtocs',func_dtoc,'number of DTOCs')
```

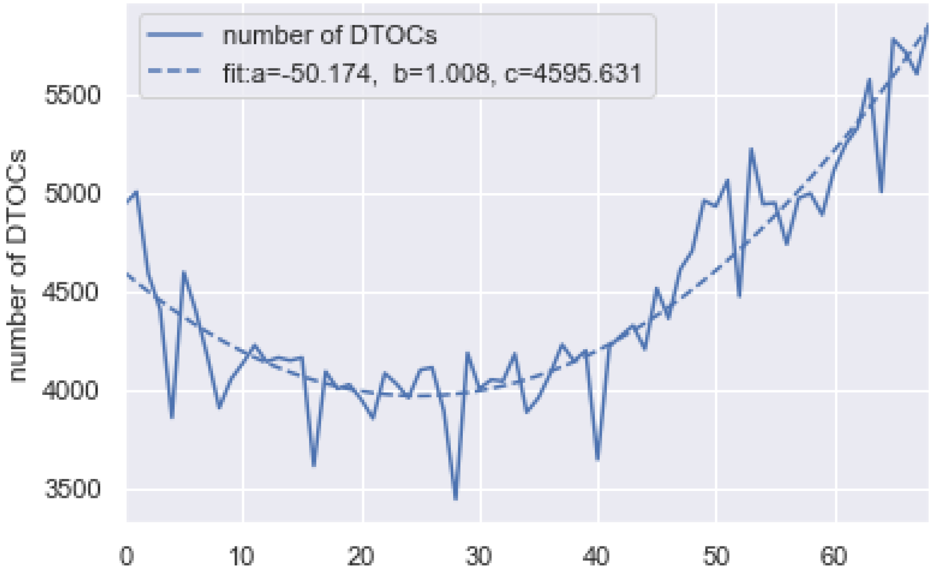

Keogh B, Monks T. *Emerg Med J* 2020; 37:95–101. doi: 10.1136/emermed-2018-207917

```
In [27]: df = detrend_using_func(df,'total_breaches',func_dtoc,'number of breaches','r')
```

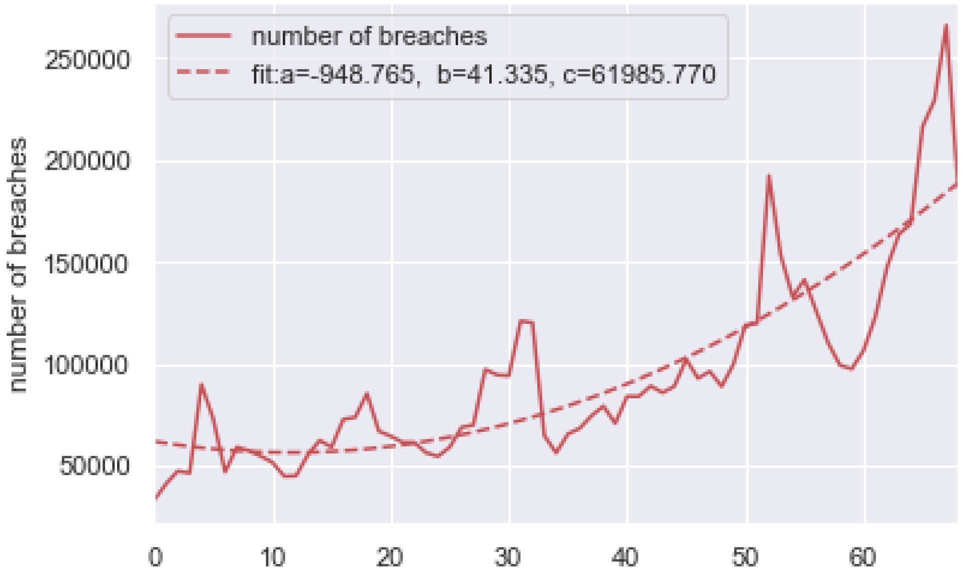

Plotting the detrended time-series (below) we see there is now no increasing trend over time.

```
In [28]: fig = plt.figure() # Create matplotlib figure
         ax = fig.add_subplot(111) # Create matplotlib axes
         ax2 = ax.twinx() # Create another axes that shares the same x-axis as ax.

         df.total_breaches_detrend.plot(color='red', ax=ax)#, width=width)#, position=1)
         df.total_dtocs_detrend.plot(color='blue', ax=ax2)

         ax.set_ylabel('Number of breaches')
         ax2.set_ylabel('Number of dtocs')
         ax2.grid(b=False)

         #sort legend
         lns = ax.get_lines()+ax2.get_lines()
         ax2.legend(lns,['breaches','DTOCs'],frameon=True)

         plt.show()
```


Keogh B, Monks T. *Emerg Med J* 2020; 37:95–101. doi: 10.1136/emermed-2018-207917

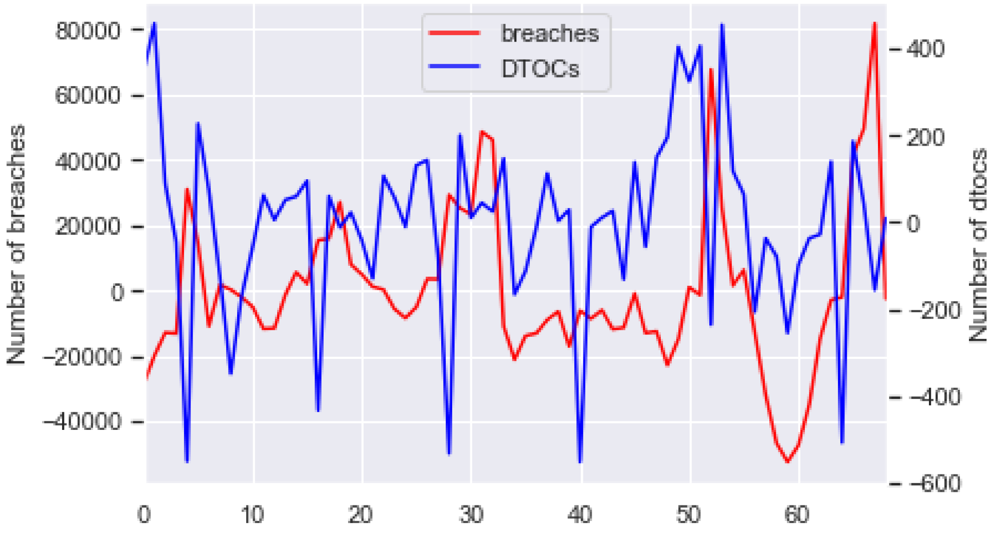

Is the data now stationary? Again using a dickey-fuller test to ascertain if detrended data is stationarity.

```
In [29]: check_stationarity(df['total_breaches_detrend'])

ADF Statistic: -3.952409
p-value: 0.001681
        1%: -3.530
        5%: -2.905
        10%: -2.590


In [30]: check_stationarity(df['total_dtocs_detrend'])

ADF Statistic: -1.586171
p-value: 0.490537
        1%: -3.551
        5%: -2.914
        10%: -2.595
```

Although we have removed the long-term trend, and breaches now appears to be stationary, it appears that the DTOCs timeseries may still not be stationary (p-value = 0.490).

Re-assesing the level of autocorrelation using the ACF we see that the seasonal variation is still present in the timeseries, but the high and persistent levels of autocorrelation over many lags has been removed.

```
In [31]: fig = sm.graphics.tsa.plot_acf(df['total_breaches_detrend'],lags=18)
```

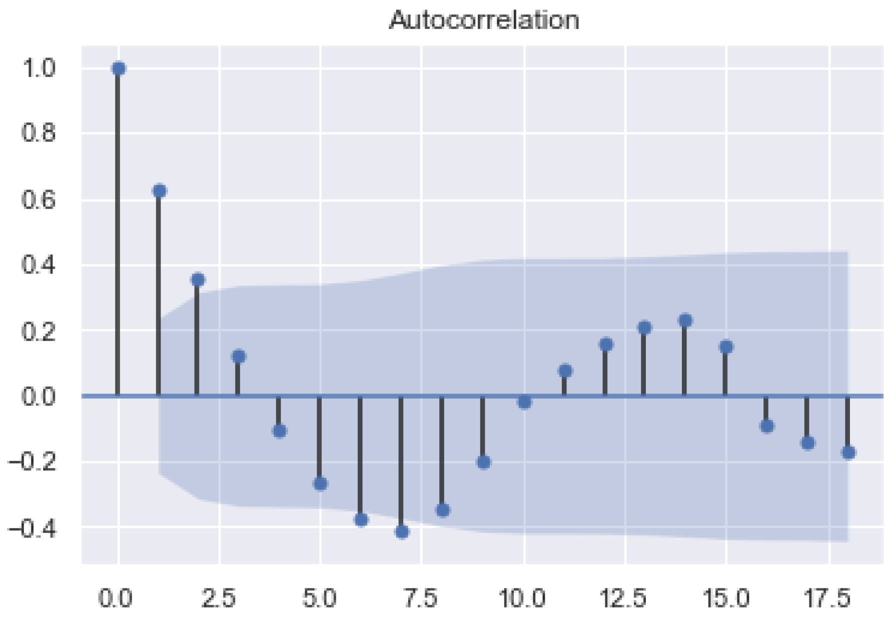


```
In [32]: fig = sm.graphics.tsa.plot_acf(df['total_dtocs_detrend'],lags=18)
```

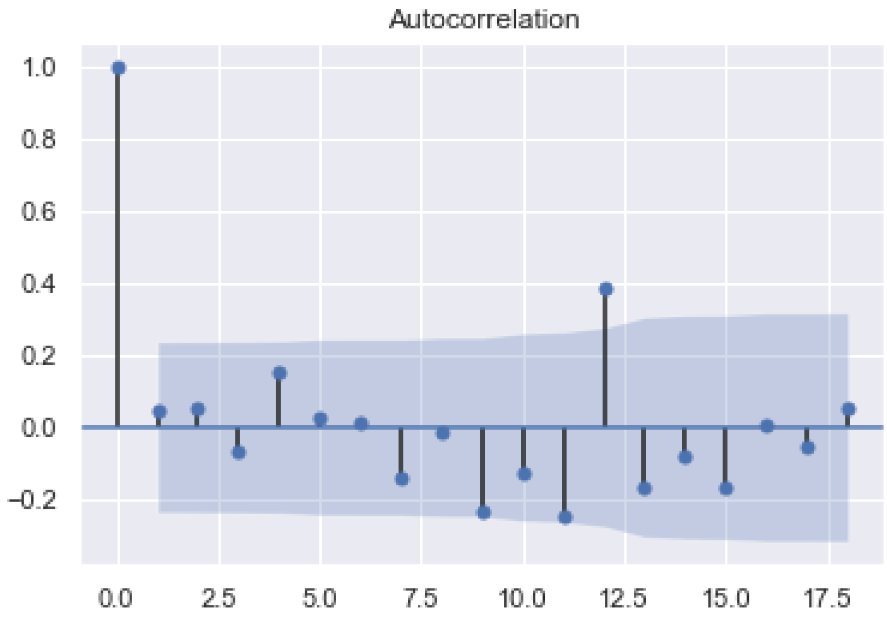

Keogh B, Monks T. *Emerg Med J* 2020; 37:95–101. doi: 10.1136/emermed-2018-207917

5 Analysis on detrended data

Completing the same linear regression analysis on the detrended data as before we find there are much smaller correlation coeficients.

```
In [33]: # get coeffs of linear fit
         slope, intercept, r_value, p_value, std_err = stats.linregress(df['total_dtocs_detrend'],df['t
```

```
In [34]: fig, ax = plt.subplots(figsize=(6,5))
         y = df['total_breaches_detrend']
         x = df['total_dtocs_detrend']
         plt.plot(x,y,'o', label='monthly data')
         plt.plot(x, intercept + slope*x, 'r', label='fitted line: R$^2$ = {0:.2f}'.format(r_value**2))
         plt.legend(frameon=True,fontsize='large')
         ax.set_xlabel('number of DTOCs')
         ax.set_ylabel('number of breaches')
         plt.show()
```

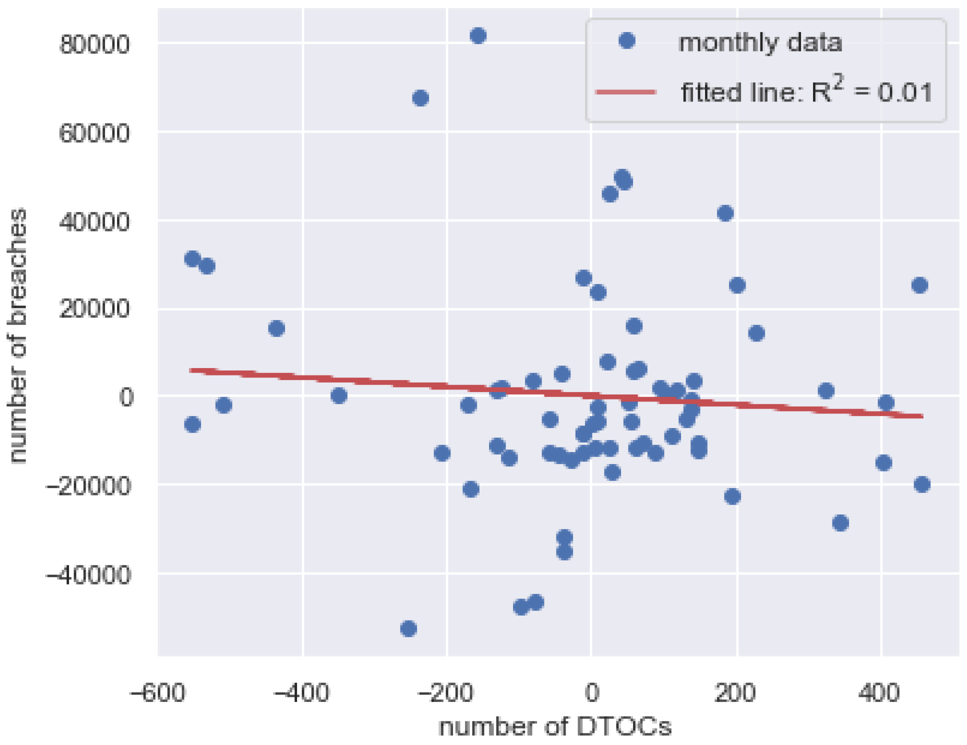


```
In [35]: df[['total_breaches_detrend','total_dtocs_detrend']].hist();
```


Keogh B, Monks T. *Emerg Med J* 2020; 37:95–101. doi: 10.1136/emermed-2018-207917

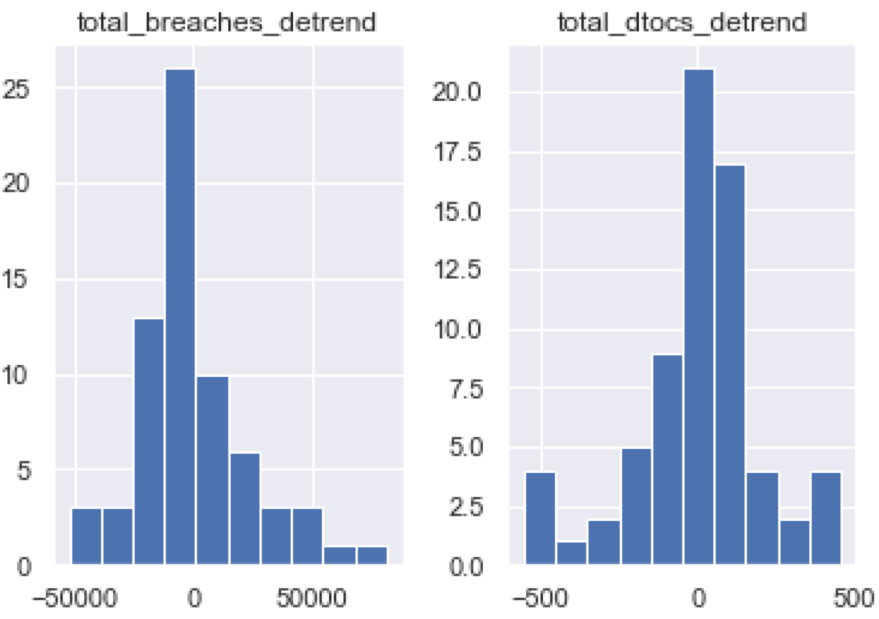


```
In [36]: xdata=df['total_breaches']
         ydata=df['total_dtocs']

         from scipy.stats.stats import spearmanr
         from scipy.stats.stats import pearsonr

         def test_corrs(xdata,ydata,test):
             result = test(xdata,ydata)
             return(result)

         def create_test_df(df):
             index =[]
             corrs = []
             ps = []

             corr,p = test_corrs(df['total_breaches'],df['total_dtocs'],pearsonr)
             index.append('original (pearson)')
             corrs.append(corr)
             ps.append(p)


             corr,p = test_corrs(df['total_breaches'],df['total_dtocs'],spearmanr)
             index.append('original (spearman)')
             corrs.append(corr)
             ps.append(p)
```


Keogh B, Monks T. *Emerg Med J* 2020; 37:95–101. doi: 10.1136/emermed-2018-207917

```
        corr,p = test_corrs(df['total_breaches_detrend'],df['total_dtocs_detrend'],pearsonr)
        index.append('detrended (pearson)')
        corrs.append(corr)
        ps.append(p)



        corr,p = test_corrs(df['total_breaches_detrend'],df['total_dtocs_detrend'],spearmanr)
        index.append('detrended (spearman)')
        corrs.append(corr)
        ps.append(p)


        result = pd.DataFrame(data={'correlations':corrs,'p_values':ps},index=index)
        return(result)

    corrs = create_test_df(df)
```

In [37]:
```
corrs.round(4) # produce table for paper
```

Out[37]:

	correlations	p_values
original (pearson)	0.7101	0.0000
original (spearman)	0.5416	0.0000
detrended (pearson)	-0.0915	0.4544
detrended (spearman)	0.0178	0.8844

The p-value roughly indicates the probability of an uncorrelated system producing datasets that have a correlation at least as extreme as the one computed from these datasets. The high p-values for the detrended data indicate that there is a high probability that breaches and DTOCs have little correlation to one another.

In [ ]:

6 Plots for paper with time highlighted in colour

In [38]:
```
#### setup figure plots
fig,ax = plt.subplots(3,1,figsize=(5,11));
VarA = 'Number of DTOCs'
VarB = 'Number of breaches'

df['Month'] = df.index;
df[VarA] = df['total_dtocs'];
df[VarB] = df['total_breaches'];


#### plot line graph
df[[VarB]].plot(ax=ax[0],color='k', linewidth=0.5);
```


Keogh B, Monks T. *Emerg Med J* 2020; 37:95–101. doi: 10.1136/emermed-2018-207917

```
df[[VarA]].plot(color='k', linewidth=0.5, linestyle=':',ax=ax[1]);

cmap='winter'

#### scatter on line plots
df[['Month',VarB]].plot('Month',VarB,kind='scatter',ax=ax[0],c='Month',colormap=cmap,colorbar=
df[['Month',VarA]].plot('Month',VarA,kind='scatter',ax=ax[1],c='Month',colormap=cmap,colorbar=
ax[0].get_legend().remove()
ax[1].get_legend().remove()


#### scatter plot
df[[VarA,VarB,'Month']].plot(VarA,VarB,kind='scatter',ax=ax[2],c='Month',colormap=cmap); #,lab
## regression
# get coeffs of linear fit
slope, intercept, r_value, p_value, std_err = stats.linregress(df['total_dtocs'],df['total_bre

print('R^2 value of data with original analysis: ', (r_value**2).round(2))

y = df['total_breaches']
x = df['total_dtocs']
plt.plot(x, intercept + slope*x, 'r', label='fitted line: R$^2$ = {0:.2f}'.format(r_value**2))
plt.legend(frameon=True,fontsize='large');

fig.tight_layout(pad=1)

plt.text(2700, 303000*3, 'a', fontsize=18)

plt.text(2700, 295000*2, 'b', fontsize=18)

plt.text(2700, 280000, 'c', fontsize=18);

# fig.savefig('Figure2',dpi=800)
```

```
R^2 value of data with original analysis:  0.5
```


Keogh B, Monks T. *Emerg Med J* 2020; 37:95–101. doi: 10.1136/emermed-2018-207917

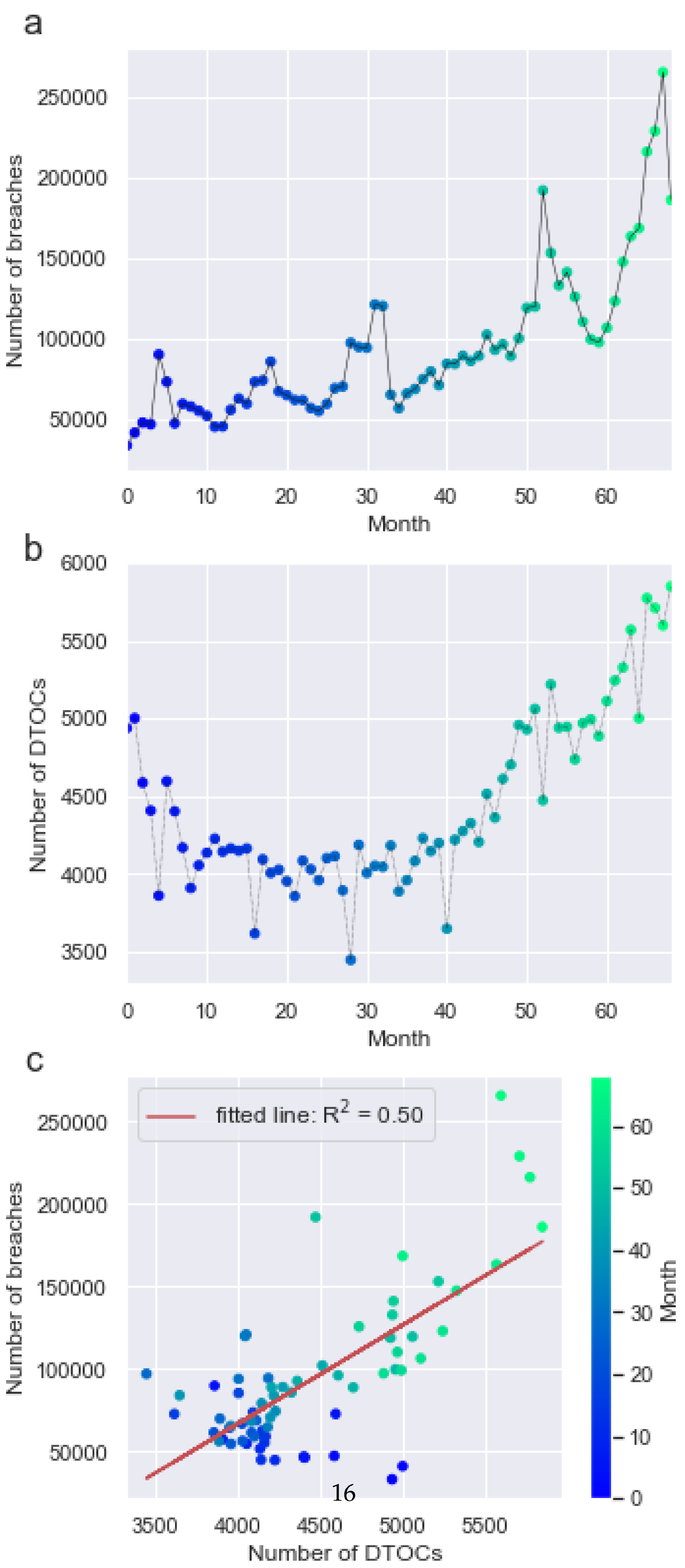
a
Number of breaches
250000
200000
150000
100000
50000
0
10
20
30
40
50
60
Month
b
Number of DTOCs
6000
5500
5000
4500
4000
3500
0
10
20
30
40
50
60
Month
c
fitted line: R2 = 0.50
Number of breaches
250000
200000
150000
100000
50000
3500
4000
4500
5000
5500
Number of DTOCs
Month
60
50
40
30
20
10
0

```
In [39]: # get coeffs of linear fit
         slope, intercept, r_value, p_value, std_err = stats.linregress(df['total_dtocs_detrend'],df['t

         print('R^2 value of data with detrended analysis: ', (r_value**2).round(2))

R^2 value of data with detrended analysis:  0.01

In [40]: fig, ax = plt.subplots(figsize=(5,4))
         y = df['total_breaches_detrend']
         x = df['total_dtocs_detrend']
         df[['total_dtocs_detrend','total_breaches_detrend','Month']].plot('total_dtocs_detrend','total

         plt.plot(x, intercept + slope*x, 'r', label='fitted line: R$^2$ = {0:.2f}'.format(r_value**2))
         plt.legend(frameon=True,fontsize='medium')
         ax.set_xlabel('Number of DTOCs')
         ax.set_ylabel('Number of breaches')
         plt.show()


         fig.tight_layout(pad=1)
         # fig.savefig('Figure3',dpi=800)
```

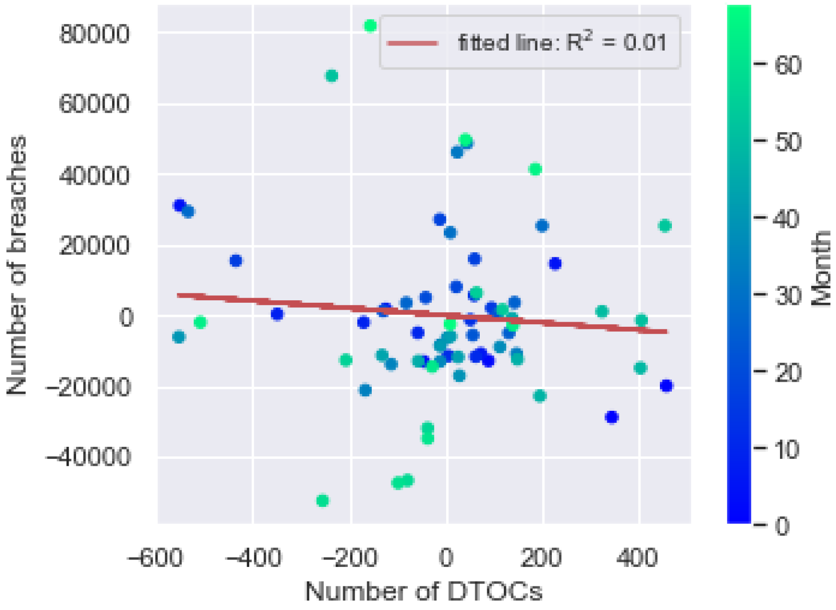


```
In [ ]:
```


Keogh B, Monks T. *Emerg Med J* 2020; 37:95–101. doi: 10.1136/emermed-2018-207917
